# Impact of maternal diabetes mellitus on fetal atrial strain

**DOI:** 10.1007/s10554-024-03194-9

**Published:** 2024-07-27

**Authors:** Faraz Pathan, Penny Lam, Shanthosh Sivapathan, Shahab Pathan, Zhiyu Gao, Sam Orde, Deva Nirthanakumaran, Kazuaki Negishi, Ralph Nanan

**Affiliations:** 1 Sydney Medical School Nepean, Charles Perkins Centre, The University of Sydney, Sydney, Australia; 2 Department of Cardiology, Nepean Hospital, Sydney, Australia; 3 Department of Perinatal Ultrasound, Christopher Kohlenberg, Nepean Hospital, Sydney, Australia; 4 Department of Intensive Care Medicine, Nepean Hospital, Sydney, Australia; 5 Department of Pediatrics, Nepean Hospital, Sydney, Australia; 6 Nepean Clinical School, University of Sydney, Level 5 South Block Derby Street, Kingwood, Australia

**Keywords:** Maternal diabetes, Fetal left atrial strain, Developmental origin of disease

## Abstract

While Maternal Diabetes Mellitus (DM) is well known to affect the size and function of multiple fetal organ systems, effects on developing heart chamber function remain difficult to assess. We sought to determine the independent impact of maternal DM on fetal cardiac function in middle pregnancy. We prospectively recruited mothers with all categories of DM and non-diabetic healthy controls (NDC). Echocardiograms were optimized for chamber quantification and strain analysis. Left atrial area (LAA), LA strain (LAS), right atrial strain (RAS), global longitudinal ventricular strain (GLS) and Right ventricular free wall strain (RV FWS) were evaluated by 2 blinded operators. After excluding 9 mothers with poor fetal image quality, images from 104 mothers with DM and 47 NDC were analyzed. Mothers with DM and NDCs were well matched for age, blood pressure, smoking prevalence, and gestational age. Fetal heart rate (FHR) was significantly higher in fetuses of mothers with DM compared to NDC (147 ± 10 bpm vs. 144 ± 8, p = 0.04). LAA in fetuses of mothers with DM trended towards being larger in size (1.68 ± 0.4cm^2^ vs. 1.56 ± 0.4cm^2^, p = 0.08). Fetal septal diameters were larger in maternal DM compared to NDC (2.7 ± 0.5 cm vs. 2.5 ± 0.5 cm, p = 0.001). GLS was similar between the groups. Fetal LAS was lower in maternal DM (28.8 ± 8.8% vs. 33.3 ± 10.4%, p = 0.007) and was independently associated with maternal DM after adjusting for GLS and FHR. Fetal RAS was lower in maternal DM (27.7 ± 10.4% vs. 31.8 ± 10.3%, p = 0.007), however only determinates were estimated fetal weight and RV FWS. Maternal DM independently impairs fetal LA function in mid pregnancy. These early functional changes in the developing heart warrant future studies investigating impact on cardiovascular health.

## Background

Maternal conditions such as diabetes mellitus (DM) can have a profound effect on the developing fetus. The prevalence of diabetes in pregnancy is predicted to stabilise at 15.8–16.0% between 2019–2045 [1]. Population based data illustrate that children of diabetic mothers have earlier onset of cardiac disease including heart failure and atrial fibrillation [2].

These late life cardiac outcomes may be a consequence of fetal developmental programming in-utero [3] or due to the direct impact of maternal and consequent fetal hyperglycaemia upon the myocardium. There is evidence to support the impact of maternal obesity and diabetes on fetal and childhood health resulting in reduced birth weight and increased risk of cardiovascular disease in the offspring. The impact of maternal diabetes on the developing myocardium is difficult to evaluate given the challenges in imaging the fetus and the short duration between organogenesis and delivery.

Studies thus far have relied on earlier markers of cardiovascular pathology. Left ventricular global longitudinal strain (LV GLS) has been studied. Aguilera et al., demonstrated impaired LV GLS in foetuses with maternal diabetes [4]. Miranda et al. demonstrated diastolic abnormalities and impairment of right ventricular GLS [5]. Aguilera and colleagues demonstrated the observed changes in LV GLS and right ventricular function persist after delivery and into infancy [6].

The impacts of maternal diabetes on the developing fetal left atrium have not been investigated. Atrial strain is a novel biomarker which has shown incremental value over and above traditional markers in detecting early atrial pathology [7]. Normal ranges and vendor differences have been shown in adults [8, 9]. Rato et al. have demonstrated the feasibility of fetal left and right atrial strain analysis [10].

Speckle tracking echocardiography or an iteration using edge detection enables assessment of left atrial function, which is less dependent on cardiac orientation and subsequently an ideal tool for assessment of cardiac function, as it is less influenced by fetal orientation within the uterus.

Feasibility of fetal left atrial strain (LAS) has been previously demonstrated [11, 12]. Diabetes is associated with impaired left atrial function, in adults [13], however, to our knowledge no such association between maternal diabetes and fetal LAS or RAS has been investigated.

We sought to determine the impact of maternal diabetes on the developing fetal left atrium. We hypothesized that maternal diabetes would impair fetal left atrial function when compared to healthy controls.

## Methods

### Study group

Patient were prospectively recruited prior to attending mandated 28-week fetal morphology scans – both mothers with maternal diabetes, and non-diabetic healthy controls.

Patients were excluded from the study if there was a history of preeclampsia, pregnancy induced hypertension, any other diseases which may impact fetal cardiac function, or inadequate fetal ultrasound images for atrial strain analysis.

The diagnosis of gestational diabetes mellitus was made in conjunction with the Australasian Diabetes in Pregnancy Society (ADIPS) Consensus Guidelines for the Testing and Diagnosis of Gestational Diabetes Mellitus in Australia of a fasting glucose ≥ 5.1 mmol/L, 1-h glucose of ≥ 10.0 mmol/L, or a 2-h glucose ≥ 8.5 mmol/L [14]^.^

The study was approved by the Health and Medical Human Ethics Research Committee at Nepean Hospital, Sydney, Australia (HERC ref No: H0015502). Fetuses found to have significant cardiac or extra cardiac abnormalities were excluded from the study so as not to confound results.

One hundred and sixty-two pregnant women were approached for recruitment, of which, 161 provided informed consent and 1 declined. Cine loop acquisition of the 4-ch heart was feasible on 160 women. 160 pregnant women (50 controls, 110 DM) were scanned, 9 were excluded due to poor image quality which was not appropriate for atrial strain analysis resulting in 104 mothers with diabetes (T1DM 9, T2DM 8, Gestational DM 87) and 47 Non-diabetic controls (NDC).

The baseline characteristics are shown in Table 1.

**Table 1:** Baseline characteristics

	DM (n = 104)	NDC (n = 47)	p
Mother			
Age (y)	31 ± 5	31 ± 5	0.7
BMI (kg/m^2^)	30.4 (25.1–34.8)	20.8 (21.4–27.4)	** < 0.001**
Weight (kg)	77 (65–93)	64 (62–68)	** < 0.001**
SBP (mmHg)	110 ± 11	107 ± 9	0.12
Smoking	8.20%	6.70%	0.90
Gestational Age (y)	28.6 (28.4–28.7)	28.6 (28.3–28.7)	0.63
Foetus/offspring			
Birth weight (g)	3352 ± 448	3372 ± 527	0.82
FHR (bpm)	147 ± 10	144 ± 8	**0.04**
EFW (g)	1298 (1216–1445)	1288 (1201–1395)	0.34

*BMI* body mass index, *bpm* beats per minute, *DM* diabetes mellitus, *EFW* estimated fetal weight, *g* grams, *kg* kilograms, *m*^*2*^ meters squared, *n* number, *NDC* no diabetic control, *y* years

### Clinical variables

We documented relevant maternal clinical variables including demographics, height, weight, body mass index (BMI), maternal parity, systolic and diastolic blood pressure (SBP, DBP) and smoking history. The following measurements were documented for the foetus: Fetal heart rate (FHR), estimated fetal weight (EFW) and birth weight.

### Fetal ultrasonography and strain analysis

The scans were performed by a single operator trained in fetal ultrasonography (PL) using a GE Voluson ultrasonography machine (General Electric Healthcare, Milwaukee, WI), with an abdominal 4–8 MHz wideband convex volume transducer (RAB6-RS). Biometric measurements of the fetal head circumference and body dimensions using standardised image planes were obtained and applied to Hadlock’s formula for an estimation of the fetal weight [15].

For echocardiographic analysis we acquired a cine 4 chamber view with a minimum of 4–5 beats. Left atrial area (LAA) and interventricular septal diameter (IVS) were measured using the 4 chamber view as previously described, and perpendicular to the plane of insonation when possible [16].

**Fig. 1:**
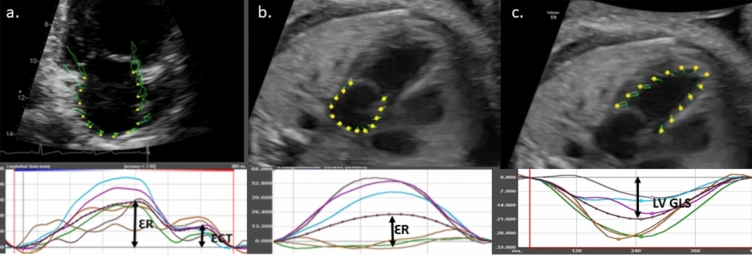
Fetal LA strain/ LV strain and Adult LA strain. Adult LA strain has a biphasic curve corresponding with ER reservoir function and ECT conduit function (**a**), Fetal LA strain due to higher heart rates have a single peak corresponding with reservoir function denoted by LAS (**b**) and fetal LV GLS is denoted by a single negative peak. Strain is a measure of relative deformation (**c**). The 6 curves correspond to 6 regions of interest. The % strain is calculated using the following operation strain = ((L − L0)/L0)* 100. In ventricular systole the left atrium enlarges, in early diastole it empties in to the left ventricle reducing in size. Finally following the p wave atrial contraction results in the atrium contracting to its smallest size. Reminder of abbreviations as per Tables 1 and 2

Atrial strain was analysed on commercial software (TomTec Image Arena, Munich, Germany). We used fetal cardiac 4-chamber (4ch) view with the onset of mitral valve closure used as the zero-reference point (R-R Gating) as has been previously described [17]. A region of interest was drawn along the LA and RA endocardial border (Fig. 1), tracking was reviewed to ensure that it was appropriate and a true representation of atrial motion. The resulting atrial strain curve provided 1 peak consistent with reservoir left atrial strain LAS (Fig. 1). We allowed exclusion of 1 segment if tracking was inadequate; if more segments were inadequate, the scan was excluded from analysis. The foramen ovale in the inter atrial septum was included both in measurements of area and function and was contoured across.

Similarly, left ventricular global longitudinal strain (LV GLS) and right ventricular free wall strain (RV FWS) assessment was performed using only the 4 ch view in keeping with standard guidelines [18]. The muscular intraventricular septum (IVS) was measured closest to the base at the time of maximum ventricular filling. The fetal position, at the time of measurement, was left or right decubitus position with the IVS. at a 90-degree angle.

Two operators (F.P. and S.S) performed strain measurements on de-identified cases, both operators were blinded to maternal health status. 5 measurements were obtained and the 3 most concordant measures of LAS were averaged. Additional parameters assessed were LAA, septal thickness (IVS) and left ventricular LV GLS (assessed as an absolute value).

### Statistical analysis

Baseline categorical variables are shown as number and percentage and computed using the Chi2 test. Normality of data was assessed using the Shapiro–Wilk test and histogram analysis. Continuous variables are presented as mean ± SD and compared using the independent samples student t test. If not normally distributed, they are presented as median and inter quartile range (IQR), and compared with the Mann–Whitney U test.

Univariable linear regression models were used to assess determinants of left and right atrial function (measured using LAS) and left atrial size (measured using LAA). Two separate multivariable models were constructed. In model 1 variables with p value < 0.1 on univariable analysis were inserted into 2 separate multivariable models for LAS and LAA. In model 2 all clinical and imaging covariates were included in separate models for LAS and LAA.

Collinearity of measures was assessed using variance inflation factor (VIF) with a VIF > 10 considered to be significant collinearity.

Inter- and intra-observer variability was computed using the Intraclass correlation coefficients (ICC) using a two-way mixed model with absolute agreement between measures. Reliability was also assessed using the coefficient of variance (COV). A p value < 0.05 was considered statistically significant. Statistical analysis was performed using MedCalc for Windows, version 15.0 (MedCalc Software, Ostend, Belgium).

## Results

There were no significant differences between control and diabetic pregnancies with respect to maternal age, blood pressure, smoking history, and gestational age at the time of fetal echocardiography. The diabetic mothers had a significantly higher BMI, median (IQR) [30.4 kg/m2 (25.1–34.8) vs. 20.8 kg/m2 (21.4–27.4), p < 0.001] and had higher weight [77 kg (65–93) vs. 64 kg (62–68), p < 0.001]. The median parity was 1 (0–1.5) for controls and 0 (0–1) for diabetic mothers (p < 0.001). FHR was higher in foetuses of diabetic mothers (147 bmp ± 10 vs144 bmp ± 8, p 0.04). All other fetal characteristics were similar (Table 1).

There was no significant difference between fetal birth weight for mothers with DM [3.35 (0.45)] and the controls [3.37 (53)]. In contrast, length of gestation was reduced in mothers with DM [38.5 weeks (IQR 38.0–39.1) vs. 39.2 (IQR 39.1–39.6)].

### Fetal echocardiography

As seen in Table 2, there was a non-statistically significant trend to larger LAA in foetuses exposed to maternal diabetes (1.68 ± 0.4 cm^2^ vs. 1.56 ± 0.4cm^2^, p = 0.08). The IVS. diameter was larger in this population (2.7 ± 0.5 cm vs. 2.5 ± 0.5 cm, p = 0.001). There was no difference in LV GLS between the two groups. The LAS was significantly lower in fetuses with diabetic mothers compared to foetuses of NDCs: 28.8% ± 8.8% vs. 33.3% ± 10.4%; p 0.007.

**Table 2:** Echocardiographic parameters

	DM	NDC	p
LAA (cm^2^)	1.68 ± 0.4	1.56 ± 0.4	0.08
RAA (cm2)	1.50 ± 0.3	1.40 ± 0.3	0.08
IVS. D (cm)	2.7 ± 0.5	2.5 ± 0.5	**0.001**
LV GLS %	15.3 ± 4.4	15.4 ± 3.9	0.88
LAS %	28.8 ± 8.8	33.3 ± 10.4	**0.007**
RAS%	27.7 ± 10.4	31.8 ± 10.3	**0.02**
RV FWS%	15.0 ± 6.6	17.0 ± 7.4	0.10
FR (hz)	41 (38–42)	34 IQR (31–38)	0.003

*FR* frame rate, *IVSD* interventricular septum diameter, *LAA* left atrial area, *LAS* left atrial strain, *LV GLS* left ventricular global longitudinal strain, *NDC* nondiabetic control, *RAA* right atrial area, *RAS* right atrial strain, *RVFWS* right ventricular free wall strain

As seen on Table 2 RAS was significantly lower in fetuses with diabetic mothers compared to foetuses of NDCs: 27.7% ± 10.4% vs. 31.8% ± 10.3%; p 0.02. Furthermore, there was no difference in RV FWS between the two groups, although there was a trend to lower RV FWS strain in fetuses with diabetic mothers (p = 0.10).

Univariable linear regression (Table 3) demonstrated that LV GLS (beta 0.43, p = 0.03) and maternal DM (beta − 4.5, p < 0.01) were determinants of LAS. FHR (beta − 0.17, p = 0.05) was borderline significant. EFW (beta 0.001, p < 0.01) was a determinant of LAA, whilst maternal DM was of borderline significance (beta 0.11, p = 0.08). All other variables were non-significant.

**Table 3:** Univariable and Multivariable regression analyses for determinants of left atrial function (LAS)

	Univariable		Multivariable Model 1		Multivariable Model 2	
	Beta	P	Beta	P	Beta	P
Maternal						
Age (y)	− 0.09	0.58			− 0.10	0.58
BMI (kg/m^2^)	0.004	0.97			0.09	0.52
Maternal DM	***− 4.5***	< 0.01*	**− 4.0**	**0.02**	**− 4.1**	**0.04**
Maternal Smoking	− 0.76	0.79			0.75	0.8
Fetal						
Fetal heart rate (bpm)	**− *0.17***	0.05*	− 0.09	0.28	− 0.15	0.12
Estimated fetal weight (/100 g)	0.88	0.84			0.88	0.25
Gestational Age (y)	− 0.55	0.61			− 2.1	0.23
Echo						
IVS. (cm)	− 1.4	0.37			− 1.22	0.49
LV GLS (%)	***0.43***	**0.03**	− 0.06	0.06	0.35	0.09

Reminder as Table 1. And Table 2. Variables which marked with (*) were included in multivariate model 1

*NS* non-significant

The multivariable model 1 (Table 3) for LAS included LV GLS, maternal DM, and FHR. Maternal DM was the only independent determinant of LAS (beta − 4.0, p = 0.02). LV GLS approached statistical significance (beta − 0.06, p = 0.06). Multivariable model 2 included all clinical and imaging covariates and only Maternal DM (beta − 4.1, p = 0.04) was a predictors of LAS.

Univariable linear regression (Table 4) demonstrated that RV FWS (beta 0.34, p = 0.004) and maternal DM (beta − 4.1, p = 0.023) and FHR (beta − 0.23, p = 0.01) were determinants of RAS. IVS. diameter (beta − 0.2.8, p = 0.10) was borderline significant. All other variables were non-significant.

**Table 4:** Univariable and Multivariable regression analyses for determinants of right atrial function (RAS)

	Univariable		Multivariable model 1		Multivariable model 2	
	Beta	P	Beta	P	Beta	P
Maternal						
Age (y)	0.19	0.27			0.1	0.56
BMI (kg/m^2^)	− 0.09	0.44			0.03	0.82
Maternal DM	***− 4.1***	**0.023**	− 3.1	0.08	− 3.2	0.12
Maternal Smoking	0.43	0.89			2.5	0.4
Fetal						
Fetal heart rate (bpm)	**− *0.23***	**0.01**	− 0.16	0.07	− 0.16	0.08
Estimated fetal weight (/100 g)	0.003	0.59			0.02	**0.03**
Gestational Age (y)	0.19	0.27			− 3.3	0.06
Echo						
IVS. (cm)	− 2.8	0.10			− 2.5	0.13
RVFWS (%)	***0.34***	**0.004**	**0.27**	**0.03**	**0.27**	**0.02**

The multivariable model 1 (Table 4) for RAS included RV FWS, maternal DM, and FHR. RV FWS (beta 0.27, p = 0.03) was the only independent determinant of RAS. Multivariable model 2 included all clinical and imaging covariates and EFW (beta 0.02, p = 0.03) and RV FWS (beta 0.27, p = 0.02) were predictors of RAS. 

Both multivariable analysis for LA size (Table 5) demonstrated that EFW was the only independent determinant of LAA (beta 0.0007 p < 0.01) with Maternal DM trending towards significance (beta 0.1, p 0.09).

**Table 5:** Univariable and Multivariable regression analyses for determinants left atrial area (LAA)

	Univariable		Multivariable Model 1		Multivariable Model 2	
	Beta	P	Beta	P	Beta	P
Maternal						
Age (y)	0.005	0.48	–		0.008	0.22
BMI (kg/m^2^)	− 0.002	0.64	–		− 0.005	0.33
Maternal DM	0.11	0.08*	0.1	0.09	0.13	0.09
Parity	0.002	0.92	–		− 0.001	0.95
Maternal smoking	0.07	0.56			0.13	0.27
Fetal						
Fetal heart rate (bpm)	− 0.002	0.60	–		− 0.003	
Estimated fetal weight (/100 g)	**0.07**	< 0.001*	**0.07**	** < 0.001**	**0.11**	** < 0.001**
Gestational Age (y)						
Echo						
IVS. (cm)	0.03	0.61	–		− 0.03	0.69
LV GLS (%)	− 0.005	0.47	–		− 0.003	0.66

### Reproducibility

The intra observer reproducibility was good with an ICC 0.89 (95% CI 0.76–0.95) and COV of 12.3%. The inter observer reproducibility was modest ICC 0.65 (95% CI 0.20–0.84) and COV of 18.8%.

## Discussion

We have conducted a study to assess fetal LAS in relation to maternal diabetes. We successfully evaluated the impact of maternal DM on fetal atrial size (LAA) and multi chamber strain. The fetal echocardiographic changes included thicker IVS, a trend towards larger LAA and impairment of fetal LAS and RAS in fetuses exposed to maternal DM.

The multivariable modelling demonstrated that maternal DM is significantly associated with lower fetal left atrial function. Furthermore, asides from an expected association between fetal size and LA size, there is a trend towards maternal diabetes increasing LA size, albeit not reaching statistical significance. As expected, mothers who were diabetic had higher BMIs, weight and their fetuses had higher FHR.

Although RA strain was lower in maternal diabetes and RV FWS trended to be lower on multivariable adjustment determinants of RAS were EFW and RV FWS.

Our study adds to previous literature on fetal strain analysis. It reproduces findings by Miranda et al. showing no significant difference in LV GLS [5], which, is different to the observation of impaired LV GLS shown by Kurlarni et al. and Aguilera et al. [4, 19]. Furthermore, our study shows a trend to reduction in RV strain, which was also demonstrated by Miranda et al. The current study evaluated LAS and RAS and added a multivariable analysis demonstrating the independent association between fetal LAS and maternal diabetes. The results suggest that in utero exposure to maternal DM may cause early functional impairment of fetal left atrial function- independent to left ventricular function. Further, our research supports the relationship between reduced fetal left atrial shortening fractions in diabetic pregnancies presented by Zielinsky et al. [20]

DM is known to result in increased systemic inflammation and oxidation, and this may impact fetal LA strain. However, we did not collect the necessary biomarkers to assess this in this study, and future research would be important to investigate this.

### Clinical implications

We have demonstrated the impact of maternal diabetes on the developing fetus in utero using a sensitive marker of cardiac function (atrial stain).

The clinical implications of these findings would suggest that maternal DM impacts LA strain, which may be an effect of atrial impairment or underlying diastolic dysfunction. These results need to be replicated and contextualized in a future study that incorporates diastology to see if the observed differences are a result of fetal diastology .

Previous assessment of fetal LAS, due to placental dysfunction did not show significant impairment of atrial function [11]. A previous study using tissue Doppler imaging and pulse wave Doppler imaging has demonstrated changes in flow patterns in maternal obesity and diabetes [21].

Maternal diabetes and resulting fetal hyperglycaemia has been shown to impact fetal diastolic function as early as the first trimester [22], with myocardial hypertrophy and an independently reduced mitral E/A ratio as illustrated by Mohsin et al [24].

The clinical relevance of this myocardial hypertrophy is unclear as it has been shown to resolve within the first few months are resolution of fetal hyperinsulinemia [24].  The resultant fetal hyperglycaemia may cause atrial and ventricular dysfunction earlier than the time necessary for overt structural changes. This parallels findings in adults where atrial function measured by LAS is impaired in diabetic patients despite normal left atrial size [23].

This impairment in cardiac function may explain the higher fetal heart rate demonstrated in our study and previously using cardiotocography [25]. The association between fetal heart rate and atrial strain suggests the mechanism by which maternal diabetes increases fetal heart rate is related to cardiac dysfunction and the need to maintain cardiac output by increasing heart rate.

The effect of maternal DM was non significant and the primary determinants on multivariable modelling were EFW and RV FWS. This may be related to the relatively modest sample size of the current study.

### Limitations

There are several limitations to this study, which include the modest sample size, moderate reproducibility, modest inter-observer variability, the lack of additional maternal baseline data and the need for alternative Doppler studies to better demonstrate changes in cardiac function. Further measures of fetal diastolic function (eg. inflow Doppler, umbilical artery, MPI) were not measured in this study, and may be of significance in future studies.

We did not find the expected difference in fetal birth weight between the two groups. However, this may be due to the observed reduced fetal gestation time in mothers with DM, as the earlier delivery may explain the lack of expected difference in fetal birth weight.

Due to the sample size being modest, the trends, which were seen in LV GLS, LA size and RV FWS and maternal diabetes, may be unmasked in a larger study with higher statistical power. Indeed, the current study can help inform sample size calculations for future studies.

Modest inter-observer agreement was noted demonstrating limited reproducibility. This may indicate that further training between the operators is required to streamline measurement techniques. We did, however, perform the cardiac functionality assessment offline, which allowed the operators FP and SS to be blinded to the maternal health status, reducing bias. Post processing image analysis also allowed the operator time to accurately review the cine loop and select the clearest segment of each clip. Although our findings demonstrate a good association with DM and cardiac function, further exploration with additional data on maternal glycated haemoglobin and blood sugar levels at the time of the scan would strengthen the results.

The study may have also benefited from an addition of tissue Doppler imaging, as there are observed significant difference in flow patterns of foetuses in NDC versus diabetes mothers [21].

The high heart rate and interplay with frame rate limited our ability to assess phasic changes in LA function as outlined by Rato et al. [10]. A limitation of this study is the lack of data regarding the angle of insonation when obtaining images, which has been shown by Semmler et al. [26], to influence left ventricular global longitudinal strain significantly, and hence can be assumed to similarly effect left atrial parameters.

We did not investigate the relationship between, or stratify the results base on, different levels of control of maternal DM. Future studies with higher sample sizes would be required to further investigate this relationship.

Further measures of fetal diastolic function (eg. inflow Doppler, umbilical artery, MPI) were not measured in this study, and may be of significance in future studies.

## Conclusion

Maternal DM may contribute to an impairment of fetal left atrial strain. There is also a trend towards maternal diabetes causing larger fetal LAA. Larger studies are needed to confirm these findings and demonstrate an association with maternal blood sugar control. Multiple time point studies are needed to confirm these findings and evaluate the legacy of the observed impairment of left atrial function.

## Abbreviations

BMI: Body mass index
COV: Coefficient of variance
DBP: Diastolic blood pressure
DM: Diabetes mellitus
EFW: Estimated fetal weight
FHR: Fetal heart rate
GLS: Global longitudinal strain
IQR: Interquartile range
IVS: Interventricular septal diameter
ICC: Intra class correlation coefficient
LAA: Left atrial area
LAS: Left atrial strain
LA: Left atrium
LV: Left ventricle
NDC: Non-diabetic healthy controls
SD: Standard deviation
SBP: Systolic blood pressure
VIF: Variance inflation factor
